# Non-resolution of non-alcoholic fatty liver disease (NAFLD) among urban, adult Sri Lankans in the general population: A prospective, cohort follow-up study

**DOI:** 10.1371/journal.pone.0224474

**Published:** 2019-10-29

**Authors:** Madunil Anuk Niriella, Anuradhani Kasturiratna, Thulani Beddage, Dileepa Senajith Ediriweera, Shamila Thivanshi De Silva, K. Ruwan Perera, Chamila Erandaka Subasinghe, S Kuleesha Kodisinghe, T. Chathura Piyaratna, Vithiya Rishikesawan, Anuradha Supun Dassanayaka, Arjuna Priyadarshin De Silva, Arunasalam Pathmeswaran, Ananda Rajitha Wickramasinghe, Norihiro Kato, Hithanadura Janaka de Silva

**Affiliations:** 1 Faculty of Medicine, University of Kelaniya, Ragama, Sri Lanka; 2 University Medical Unit, Colombo North Teaching Hospital, Ragama, Sri Lanka; 3 National Center for Global Health and Medicine, Toyama, Shinjuku-ku, Tokyo, Japan; Auburn University, UNITED STATES

## Abstract

**Background:**

There are few studies investigating the natural course of non-alcoholic fatty liver disease (NAFLD) in the community. We assessed resolution of NAFLD in a general population cohort of urban Sri Lankans adults.

**Methods:**

Participants were selected by age-stratified random sampling from electoral lists. They were initially screened in 2007 and re-evaluated in 2014. On both occasions structured interview, anthropometric-measurements, liver ultrasonography, and biochemical/serological tests were performed. NAFLD was diagnosed on ultrasound criteria for fatty liver, safe-alcohol consumption (<14-units/week for men, <7-units/week for women) and absence of hepatitis B/C markers. Non-NAFLD was diagnosed on absence of any ultrasound criteria for fatty liver and safe-alcohol consumption. Resolution of NAFLD was defined as absence of ultrasound criteria for fatty liver. Changes in anthropometric indices [Weight, Body-Mass-Index (BMI), waist-circumference (WC), waist-hip ratio (WHR)], clinical [systolic blood pressure (SBP), diastolic blood pressure (DBP)] and biochemical measurements [Triglycerides (TG), High Density Lipoprotein (HDL), Total Cholesterol (TC), HbA1c%] at baseline and follow-up were compared.

**Results:**

Of the 2985 original study participants, 2148 (71.9%) attended follow-up after 7 years. This included 705 who had NAFLD in 2007 and 834 who did not have NAFLD in 2007. Out of 705 who had NAFLD in 2007, 11(1.6%) changed their NAFLD status due to excess alcohol consumption. After controlling for baseline values, NAFLD patients showed significant reduction in BMI, weight, WHR, HDL and TC levels and increase in HbA1c levels compared to non-NAFLD people. Despite this, none of them had complete resolution of NAFLD.

**Conclusion:**

We did not find resolution of NAFLD in this general population cohort. The observed improvements in anthropometric, clinical and biochemical measurements were inadequate for resolution of NAFLD.

## Introduction

Non-alcoholic fatty liver disease (NAFLD) is probably the commonest chronic liver disease worldwide. Its global prevalence is estimated to be 24% [1]. Lifestyle modification (LSM), including a hypocaloric diet, regular physical exercise, and sustained weight loss, is the corner-stone of management of all patients with NAFLD and the more aggressive form of the disease, non-alcoholic steatohepatitis (NASH) [2]. However, sustained weight loss with LSM is difficult to achieve in the long-term. Even patients who reach weight loss targets seem unable to sustain the changes over time. Because of this, repeated counselling for a healthy, hypo-caloric diet and regular physical exercise are recommended for patients with NAFLD/NASH to achieve and maintain weight loss goals [2]. A greater degree of weight loss is associated with more improvements in histopathology in NAFLD/NASH; a weight loss of 10% or more is associated with at least some improvement in all histopathological features of NASH, including portal inflammation and even fibrosis [3].

Although many studies on NAFLD incidence and remission are available, there are very few studies investigating its course in a general population employing prospective cohort follow-up study methodology. The Dionysos study reported a 50% remission rate of fatty liver in 336 persons after a follow-up period of 8.5 years, with ethanol intake the only risk factor for the decrease in the rate of remitting of fatty livers [4]. Zelber-Sagi et al reported a 36% resolution rate in 66 Israeli patients with NAFLD after 7 years, with a 75% remission rate among NAFLD patients who lost 5% or more of their baseline weight [5]. A more recent general population study from China reported a 24.6% remission rate in 134 individuals with NAFLD after 6 years; those with lower weight at baseline and male subjects were more likely to undergo NAFLD remission [6].

We selected subjects who participated in the Ragama Health Study (RHS), an ongoing community-based cohort follow-up study on non-communicable diseases in Sri Lanka [7], to investigate resolution of NAFLD after 7 years follow up. The RHS consists of adults selected randomly from an urban general population. The cohort has a high prevalence of the components of metabolic syndrome and obesity [7], and a NAFLD prevalence of 32.6% and annual incidence rate of 6.2% [7, 8]. Prevalent and incident NAFLD were strongly associated with components of the metabolic syndrome and obesity, and PNPLA3 gene polymorphisms [7–9].

## Methods

The study population was originally selected by age-stratified random sampling from electoral lists of the Medical Officer of Health area, Ragama, Sri Lanka. They were screened initially in 2007 (aged 35–64 years) and invited for re-evaluation after 7 years in 2014 (aged 42–71 years) [4]. On both occasions they were subjected to a structured interview which included assessment of the level of physical activity and details of the diet, measurement of anthropometric indices, liver ultrasonography, and biochemical and serological tests.

On both occasions height was measured using a wall-mounted stadiometer to the nearest 0.1 cm. Weight was measured (in light indoor clothing) using a digital scale to the nearest 0.1 kg. Waist circumference (WC) was measured at the midpoint between the inferior border of the ribcage and the superior iliac crest. Hip circumference (HC) was measured at the level of the trochanters. WC and HC were measured in expiration to the nearest 0.1 cm using an inelastic measuring tape. Blood pressure was measured from the right upper limb in the sitting position, after rest, using an automatic blood pressure monitor (Omron 705CP, Omron Healthcare, Lake Forrest, Illinois, United States) and mean value of two readings taken five minutes apart was recorded.

Subjects were requested to present after a 12-hour fast on both occasions. A 10-mL sample of venous blood was obtained from each participant. This was used to determine fasting glucose, glycosylated haemoglobin A1c (HbA1c), serum lipids and serum alanine aminotransferase activity (ALT). All subjects underwent ultrasonography of the liver with a 8-MHz probe (Toshiba Ultrasound Diagnostic Systems SSA-51 OA, Toshiba Medical Systems Corporation, Otawara-City, Tochigi-prefecture, Japan) in 2007 and with a 5-MHz probe (MindrayDP-10 Ultrasound Diagnostic Systems, Mindray Medical International Limited, Shenzhen, China) in 2014. Ultrasonographic examination was carried out by doctors with special training in liver ultrasonography on both occasions. All subjects who had an abnormal liver on ultrasound were screened for hepatitis B and C [hepatitis B surface antigen (HBsAg) and anti-hepatitis C virus antibodies (anti-HCV) using CTK Biotech ELISA kits].

NAFLD was diagnosed on established ultrasound (US) criteria for fatty liver, safe alcohol consumption (<14 units/week for men, <7 units/week for females) and absence of hepatitis B and C markers [7]. Those without NAFLD were diagnosed on absence of any ultrasound criteria for fatty liver and safe-alcohol consumption.

Those with an initial diagnosis of NAFLD in 2007 were encouraged to adopt healthy life style modifications and lose weight at the inception, and referred for medical care for associated metabolic risk factors such as diabetes, hypertension and dyslipidemia, as appropriate. They were periodically invited to attend a community clinic for reinforcement of healthy lifestyle advice.

Resolution of NAFLD was defined by absence of US criteria for NAFLD on repeat imaging. Anthropometric indices [Weight, body mass index (BMI), WC, waist hip ratio (WHR)], clinical [systolic blood pressure (SBP), diastolic blood pressure (DBP)] and biochemical measurements [Triglycerides (TG), High Density Lipoprotein (HDL), Total Cholesterol (TC), HbA1c percentage] at baseline and follow-up were compared using the paired t test. Reported physical activity levels (MET-minutes/ week) at baseline and follow-up were compared using the Wilcoxon Sign Rank test. Differences in follow up values between NAFLD patients and people without NAFLD were assessed using ANCOVA after controlling for the baseline values. Statistical analysis was conducted using R programming language version 3.5.1.

Ethical approval for the study was obtained from the Ethics Review Committees of the Faculty of Medicine, University of Kelaniya. Informed written consent was obtained from all participants.

## Results

**Fig 1:**
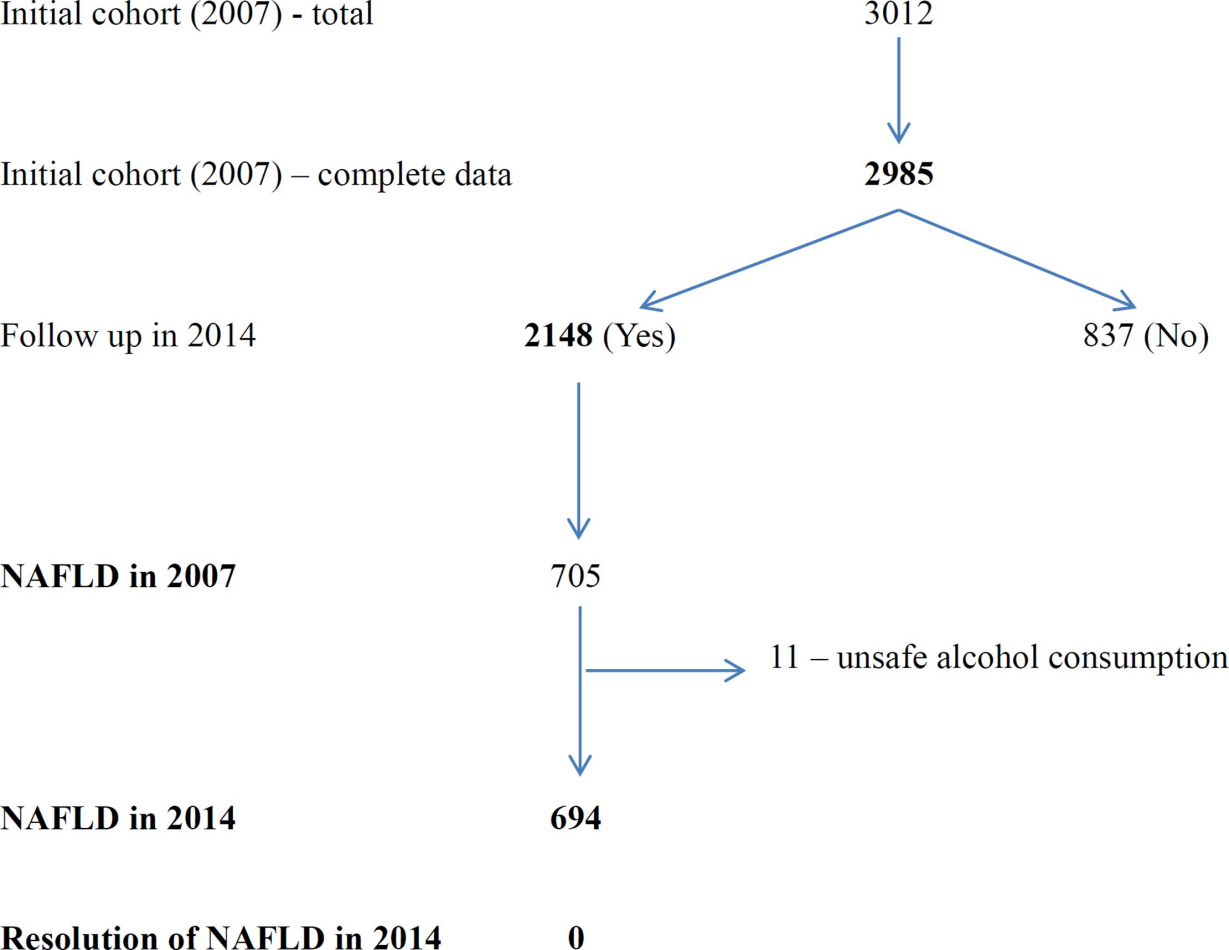
Study population [at baseline (2007) and follow-up (2014)].

Of the 2985 original study participants with complete data in 2007, 974 (32.6%) had NAFLD. 2148 (71.9%) accepted our invitation and attended follow-up after 7 years. Except for fewer males attending follow up, rest of the characteristics were similar among the inception (2007) and follow up (2014) cohorts (S1 Table) (8). Of those who attended follow-up in 2014, there were 705 [469 (66.5%) women, mean age 59.6 years [SD, 7.3]) who had been diagnosed as NAFLD in 2007 and 834 [472 (66.7%) women, mean age 59.9 [SD, 7.3]) who did not have NAFLD in 2007. During the 7 years, 11 (1.6%) of the 705 individuals changed their NAFLD status due to excess alcohol consumption (Fig 1). Therefore, 694 individuals with NAFLD at baseline with continued no or safe alcohol use were selected for assessment. The remaining 620 participants (out of the 2184) not included in the analysis were those with 1/3 ultrasound criteria for NAFLD at baseline (*indeterminate* NAFLD), unsafe alcohol users at baseline and those who had changed from safe to unsafe alcohol use during follow up.

At follow-up, those with NAFLD in 2007 showed a significant increase in the HbA1c percentage and, significant but small, reductions in some anthropometric (weight 1.6%, WC 2.2%, WHR 5.2%), clinical (SBP 2.2%) and biochemical (TG 11.9%, HDL 2%, TC 11.4%) measurements (Table 1). Despite this, none of them had ultrasonic resolution of NAFLD.

**Table 1:** Change in clinical characteristics in those with NAFLD in 2007 (baseline) and presented for re-evaluation in 2014 (follow up) [n = 694].

Variable (SD)	Baseline	Follow up	Difference (Follow up-baseline) mean (SD)	% difference	P value
BMI (kg/m^2^)	27.18 (3.78)	27.06 (4.20)	-0.12 (2.06)	-0.4	0.127
Weight (kg)	66.75 (10.60)	65.69 (11.38)	-1.06 (5.09)	-1.6	<0.001
WC (cm)	92.93 (8.49)	90.90 (9.54)	-2.03 (7.33)	-2.2	<0.001
WHR	0.96 (0.07)	0.91 (0.13)	-0.05 (0.13)	-5.2	<0.001
Systolic BP (mmHg)	141.24 (21.13)	138.10 (20.12)	-3.14 (18.55)	-2.2	<0.001
Diastolic BP (mmHg)	83.16 (11.63)	82.94 (11.20)	-0.22 (11.08)	-0.3	0.597
HbA1c (%)	6.09 (1.37)	7.09 (2.56)	1.00 (2.37)	16.4	<0.001
Triglycerides (mg/dl)	149.11 (67.8)	131.41 (58.41)	-17.70 (59.76)	-11.9	<0.001
HDL Cholesterol (mg/dl)	49.28 (4.6)	48.29 (1.6)	-0.99 (4.65)	-2.0	<0.001
Total Cholesterol (mg/dl)	213.44 (42.6)	189.05 (45.07)	-24.40 (46.41)	-11.4	<0.001
ALT (IU/L)	56.84 (27.3)	56.75 (27.0)	-0.09 (23.76)	-0.16	0.53
Physical activity MET-minutes/week median (IQ range)	3306 (760.5–8316)	2772 (594–7200)	-115.5 (8473)	-3.5	0.144

BMI- Body mass index, WC- Waist circumference, WHR- Waist hip ratio, MET–Metabolic Equivalent, SD–standard deviation

At follow up, those without NAFLD in 2007, showed significant increases in some anthropometric (BMI 3.0%, weight 2.1%, WC 0.7%), clinical (DBP 3.6%) and biochemical (HbA1c 13.3% and TG 4.9%) measurements. They also showed significant reductions in WHR, HDL, TC, ALT levels and physical activity levels over the follow up period (Table 2).

**Table 2:** Change in clinical characteristics in those without NAFLD in 2007 (baseline) and presented for re-evaluation in 2014 (follow up) [n = 834].

Variable (SD)	Baseline	Follow up	Difference (Follow up-baseline) mean (SD)	% difference	P value
BMI (kg/m^2^)	22.1 (3.46)	22.77 (3.72)	0.66 (2.01)	2.99	<0.001
Weight (kg)	54.73 (10.03)	55.85 (10.62)	1.12 (4.9)	2.05	<0.001
WC (cm)	79.99 (9.59)	80.58 (10.27)	0.59 (7.37)	0.74	0.016
WHR	0.91 (0.07)	0.88 (0.09)	-0.03 (0.09)	-3.81	<0.001
Systolic BP (mmHg)	129.56 (21.38)	129.68 (21.87)	0.12 (18.37)	0.09	0.841
Diastolic BP (mmHg)	75.86 (12.12)	78.59 (12.2)	2.73 (11.11)	3.6	<0.001
HbA1c (%)	5.6 (1.19)	6.35 (1.32)	0.74 (1.16)	13.29	<0.001
Triglycerides (mg/dl)	112.43 (60.99)	117.96 (55.45)	5.53 (59.31)	4.92	0.022
HDL Cholesterol (mg/dl)	49.58 (4.51)	48.61 (1.41)	-0.97 (4.64)	-1.96	<0.001
Total Cholesterol (mg/dl)	208.06 (40.37)	197.41 (40.46)	-10.65 (43.23)	-5.12	<0.001
ALT (IU/L)	41.45 (19.39)	23.95 (14.45)	-17.5 (19.66)	-42.22	<0.001
Physical activity MET-minutes/week median (IQ range)	4452 (1386–10572)	3846 (693–9093)	-606 (10367)	-13.6	0.003

BMI- Body mass index, WC- Waist circumference, WHR- Waist hip ratio, MET–Metabolic Equivalent, SD–standard deviation

After controlling for baseline values, NAFLD patients showed significant reduction in BMI, weight, WHR, HDL and TC levels and increase in HbA1c levels compared to non-NAFLD people (Table 3).

**Table 3:** Comparison of change in clinical characteristics in those with NAFLD [n = 694] and without NAFLD [n = 834] in 2007 (baseline) presented for re-evaluation in 2014 (follow up).

Variable (SD)	NAFLD		Non-NAFLD		P value*
	Difference (Follow up-baseline) mean (SD)	% difference	Difference (Follow up-baseline) mean (SD)	% difference	
BMI (kg/m^2^)	-0.12 (2.06)	-0.4	0.66 (2.01)	2.99	<0.001
Weight (kg)	-1.06 (5.09)	-1.6	1.12 (4.9)	2.05	<0.001
WC (cm)	-2.03 (7.33)	-2.2	0.59 (7.37)	0.74	0.545
WHR	-0.05 (0.13)	-5.2	-0.03 (0.09)	-3.81	0.016
Systolic BP (mmHg)	-3.14 (18.55)	-2.2	0.12 (18.37)	0.09	0.220
Diastolic BP (mmHg)	-0.22 (11.08)	-0.3	2.73 (11.11)	3.6	0.567
HbA1c (%)	1.00 (2.37)	16.4	0.74 (1.16)	13.29	<0.001
Triglycerides (mg/dl)	-17.70 (59.76)	-11.9	5.53 (59.31)	4.92	0.205
HDL Cholesterol (mg/dl)	-0.99 (4.65)	-2.0	-0.97 (4.64)	-1.96	<0.001
Total Cholesterol (mg/dl)	-24.40 (46.41)	-11.4	-10.65 (43.23)	-5.12	<0.001
ALT (IU/L)	-0.09 (23.76)	-0.16	-17.5 (19.66)	-42.22	0.921
Physical activity MET-minutes/week median (IQ range)	-115.5 (8473)	-3.5	-606 (10637)	-13.6	0.027

*Statistical method used—ANCOVA; BMI- Body mass index, WC- Waist circumference, WHR- Waist hip ratio, MET–Metabolic Equivalent, SD–standard deviation

## Discussion

We did not find any resolution of NAFLD after 7 years in this prospectively followed-up general population cohort known to have a high prevalence and incidence of NAFLD, metabolic syndrome and obesity [7, 8]. The observed improvements in some anthropometric, clinical and biochemical measurements, though statistically significant, were inadequate for resolution of NAFLD. To our knowledge this is the first such study in a general South Asian population.

Individuals with and without NAFLD in 2007 showed reductions of WHR, HDL, TC and physical activity and increase in HbA1c levels over the follow up period. However, only those with NAFLD showed reduction in BMI, weight, WC, SBP, DBP and TG levels over the follow up. This observed reduction is unlikely to be caused by the regression towards the mean during the follow up as the individuals without NAFLD did not show similar changes for the same variables at follow up.

The observed increases at follow up in BMI, weight, WC, DBP, TG, and the greater reduction in physical activity among those without NAFLD at baseline, compared to those with NAFLD at baseline is interesting. This may be due to people without NAFLD at baseline not having any input for lifestyle modification during the follow up period compared to those with NAFLD at baseline, who had some input, even if this was not a formal, intensive and personalized life-style intervention program.

We defined fatty liver using strict US criteria–at least 2 out the three criteria, namely, increased hepatic echogenicity compared to the spleen or the kidney, blurring of liver vasculature and deep attenuation of the ultrasonographic signal [10]. This may have selected individuals with moderate to severe fatty liver [11, 12]. Resolution was defined as the absence of any US criteria for fatty liver, which would have required an individual to reduce their hepatic fat content to less than 30% [10]. In the absence of any individuals fulfilling this criterion, it is fair to assume that those who had NAFLD at baseline continued to have at least >30% of fat content in the liver after 7 years of follow-up.

Our results are different to the few other, smaller, general population studies in the three Western and Chinese population cohorts which reported NAFLD resolution rates of about 25% to 50% [4–6]. Whether lifestyle interventions were made in these populations is unclear. The reasons for our results being different from these studies may include our selection of individuals with more severe degrees of fatty liver, a general population sample of urban, aging adults with a high prevalence of components of metabolic syndrome and obesity [7], poor adherence to lifestyle modification, as evidenced by the lack of improvement in physical activity levels, resulting in suboptimal weight loss (<2% of weight at baseline), and possible racial and genetic differences in the populations. Sri Lankan diets are high in carbohydrate content, but we could not assess changes in dietary habits in our cohort as details were incomplete and appeared unreliable–this is a limitation of our study.

Clinical evidence strongly supports the role of lifestyle modification in the management of NAFLD and NASH [13]. Weight loss decreases cardiovascular and metabolic risk in addition to regression of the liver disease. Weight reductions of ≥10% can induce a near universal resolution of non-alcoholic steatosis and steatohepatitis. It may also result in improvement of fibrosis by at least one stage. However, modest weight loss (>5%) can also produce important benefits on the components of the NAFLD, particularly steatosis [13]. In order to achieve and sustain even this modest weight loss, aggressive, motivational, dietician-led life style modification with calorie restriction and regular exercise is necessary. This type of intensive intervention was not within the scope of our study, although participants with NAFLD were referred for medical care for associated metabolic risk factors such as diabetes, hypertension and dyslipidemia, as appropriate. We also invited individuals with NAFLD to periodically visit a community-based clinic for reinforcement of healthy lifestyle practices to achieve weight loss. However, attendance at these clinics was not satisfactory. Our results show that physical activity levels had actually reduced after seven years, and there was worsening of HbA1c levels. The improvements in some metabolic associations and the small reduction in weight (1.6% from baseline) after 7 years were inadequate for NAFLD resolution. In the Chinese study by Wu et al mentioned previously, male gender and lower BMI level at baseline were associated with NAFLD remission [6]. However, there was no significant difference in the change in weight during follow-up between the sustained NAFLD and NAFLD remission groups [6].

Our study had several strengths. It was a relatively large, prospective, general population cohort follow-up study. The data collected prospectively at baseline in 2007 and at follow up in 2014 were robust and, with the exception of details on diet, complete. More than 70% of study participants (overall and individuals with NAFLD) attended follow-up after 7 years.

There were several limitations to our study. The inter-observer variability of ultrasound in the diagnosis of fatty liver was not assessed either in the initial or in the follow-up cohorts. This limitation was minimized by employing trained ultrasound operators on both occasions. Information on alcohol consumption was based only on self-reporting. This may have led to under-reporting with consequent overestimation of NAFLD [8]. Furthermore, details on diet were incomplete and were considered too unreliable for analysis. Although we invited individuals with NAFLD to periodically visit a community-based clinic, repeat attendance at these clinics was not satisfactory. We were also unable to accurately capture the number and intervals between visits to the community clinics by individual participants.

In conclusion, we did not find resolution of NAFLD after seven years in this aging, urban, adult general population known to have a high prevalence of NAFLD, metabolic syndrome and obesity. The reductions in weight and WC achieved during follow-up were inadequate for resolution of NAFLD, and more intense, sustained, personalized lifestyle interventions are necessary to achieve greater improvements in anthropometric measurements and NAFLD resolution.

## Supporting information

S1 Table Profile of the study population (8). (DOCX)

## Abbreviations

NAFLD: Non-alcoholic fatty liver disease
NASH: Non-alcoholic steatohepatitis
RHS: Ragama Health Study
US: ultrasound
BMI: body mass index
WC: waist circumference
HC: Hip circumference
HbA1c: glycosylated haemoglobin A1c
ALT: alanine aminotransferase
HBsAg: hepatitis B surface antigen
WHR: waist hip ratio
SBP: systolic blood pressure
DBP: diastolic blood pressure
TG: Triglycerides
HDL: High Density Lipoprotein
TC: Total Cholesterol
