# Supplementary material for: Non-resolution of non-alcoholic fatty liver disease (NAFLD) among urban, adult Sri Lankans in the general population: A prospective, cohort follow-up study
Source: PLoS One. 2019 Oct 29;14(10):e0224474. doi: 10.1371/journal.pone.0224474 (PMC6818953; doi:10.1371/journal.pone.0224474)
Supplement: S1 Table — (DOCX) [file pone.0224474.s001.docx]

**Supplementary material**

**S1 Table - Profile of the study population (8)**

|  | **Initial cohort 2007** | **Attended follow-up 2014** | **Did not attend follow-up 2014** |
| --- | --- | --- | --- |
|  | ***n=2985*** | ***n=2148*** | ***n=837*** |
| **Males (%)** | 1349 (45.2) | 910 (42.4)* | 439 (52.4)* |
| **Mean age (SD)** | 52.4 (7.8) | 52.4 (7.7) | 52.5 (8.1) |
| **Mean BMI (SD)** | 24.1 (4.2) | 24.3 (4.1) | 23.7 (4.4) |
| **Mean waist-hip ratio (SD)** | 0.9 (0.1) | 0.9 (0.1) | 0.9 (0.1) |
| **DM (raised FBS) [%]** | 709 (23.8) | 477 (22.2) | 232 (27.7) |
| **HBP (SBP>140, DBP>90) [%]** | 1820 (60.4) | 1298 (60.4) | 522 (62.3) |
| **Mean TG (SD)** | 131.6 (68.2) | 130.8 (68.0) | 133.5 (68.6) |
| **Mean HDL (SD)** | 49.6 (4.5) | 49.6 (4.5) | 49.6 (4.4) |
| **Mean LDL (SD)** | 136.2 (37.8) | 136.5 (37.7) | 135.3 (38.0) |

*Z=4.97; p<0.001 (Z test comparing two proportions); SD – standard deviation
